# Receptor Activated Ca^2+^ Release Is Inhibited by Boric Acid in Prostate Cancer Cells

**DOI:** 10.1371/journal.pone.0006009

**Published:** 2009-06-23

**Authors:** Kimberly Henderson, Salvatore L. Stella, Sarah Kobylewski, Curtis D. Eckhert

**Affiliations:** 1 Molecular Toxicology, School of Public Health, University of California Los Angeles, Los Angeles, California, United States of America; 2 Neurobiology, Geffen School of Medicine, University of California Los Angeles, Los Angeles, California, United States of America; 3 Environmental Health Sciences, School of Public Health, University of California Los Angeles, Los Angeles, California, United States of America; University of Hong Kong, Hong Kong

## Abstract

**Background:**

The global disparity in cancer incidence remains a major public health problem. We focused on prostate cancer since microscopic disease in men is common, but the incidence of clinical disease varies more than 100 fold worldwide. Ca^2+^ signaling is a central regulator of cell proliferation, but has received little attention in cancer prevention. We and others have reported a strong dose-dependent reduction in the incidence of prostate and lung cancer within populations exposed to boron (B) in drinking water and food; and in tumor and cell proliferation in animal and cell culture models.

**Methods/Principal Findings:**

We examined the impact of B on Ca^2+^ stores using cancer and non-cancer human prostate cell lines, Ca^2+^ indicators Rhod-2 AM and Indo-1 AM and confocal microscopy. In DU-145 cells, inhibition of Ca^2+^ release was apparent following treatment with Ringers containing RyR agonists cADPR, 4CmC or caffeine and respective levels of BA (50 µM), (1, 10 µM) or (10, 20, 50,150 µM). Less aggressive LNCaP cancer cells required 20 µM BA and the non-tumor cell line PWR1E required 150 µM BA to significantly inhibit caffeine stimulated Ca^2+^ release. BA (10 µM) and the RyR antagonist dantroline (10 µM) were equivalent in their ability to inhibit ER Ca^2+^ loss. Flow cytometry and confocal microscopy analysis showed exposure of DU-145 cells to 50 µM BA for 1 hr decreased stored [Ca^2+^] by 32%.

**Conclusion/Significance:**

We show B causes a dose dependent decrease of Ca^2+^ release from ryanodine receptor sensitive stores. This occurred at BA concentrations present in blood of geographically disparate populations. Our results suggest higher BA blood levels lower the risk of prostate cancer by reducing intracellular Ca^2+^ signals and storage.

## Introduction

One of the ways cells respond to environmental stimuli is by opening channels between sites of stored calcium, such as the endoplasmic reticulum (ER), Golgi, and mitochondria (mt), which contain high free Ca^2+^ concentrations (500 µM), and the cytoplasm, which contains low free Ca^2+^ concentrations (100 nM) [1]. A rapid rise in cytoplasmic Ca^2+^ can be achieved by capacitative calcium entry (CCE) involving release of stored Ca^2+^ by the ryanodine receptor (RyR) and the IP_3_ receptor (IP_3_R) into the cytoplasm followed by an influx of extracellular Ca^2+^. CCE activates Ca^2+^ binding proteins that regulate numerous cell functions including gene transcription, cell proliferation, vesicle secretion, and apoptosis [2], [3]. Cytoplasmic Ca^2+^ concentrations are returned to normal as Ca^2+^ is removed by transporters such as the Na^2+^- Ca^2+^ exchanger in the plasma membrane, the sarcoplasmic endoplasmic ATPase (SERCA) in the ER membrane, Ca^2+^ uniporter in mitochondria, and by binding to high affinity binding proteins [4], [5]. In this report we present evidence that physiological levels of boric acid (BA) inhibit stored Ca^2+^ release from RyR agonist sensitive sites.

Boron (B) is the 9^th^ most abundant element in seawater (425 µM B) and until recently dismissed as biologically irrelevant [6]. B is bound to oxygen in nature and in physiological fluids 98.4% is present in the form of B(OH)_3_ boric acid and 1.6% as B(OH)_4_^−^ borate. Biology has used this element in the structure of several molecules including: antibiotics in fungi [7]; quorum sensing auto inducer 2 in bacteria [8]; and the rhamnogalacturonan-II dimer in plants [9]. In plants, B is required for cell elongation, flowering and seed formation and is an integral component of food crops. Non-charged BA crosses the plasma membrane of root epidermal cells into the cytosol by passive diffusion and this is facilitated by NIP5; 1 transporters [10]. BA is partially converted to borate in the cytosol (pKa 9.6) and transported into the xylem using the export transporter BOR1 [11], [12]. A human homolog of BOR1 named NaBC1has been reported to be present in mammalian cell lines and to enhance cell proliferation at low BA concentrations [13], but this has yet to be confirmed by another laboratory. The effect of BA on growth and cell proliferation in trout, zebrafish and the mammalian HEK and HeLa cells follows an inverted U-shape with higher concentrations causing cell growth inhibition [13]–[15]. Concentrations of 60 to 100 µM BA inhibit cell proliferation in prostate cancer cells whereas high concentrations of 500 to 1000 µM BA are required to inhibit the proliferation of non-tumor prostate epithelial cells over the same time frame [16].

Human blood levels of BA reflect the local geology, water quality, and plants in the diet with a world-wide range from 2 to 120 µM (21 to 1232 ng B/g wet blood) in free living healthy men and women [17], [18]. Several human studies have observed a decrease in the risk of prostate and lung cancer, and abnormal cervical cytopathology in proportion to the amount of B ingested from food and water [19]–[22]. One study did not observe a protective effect, but differed from other studies in using a different B food database and estimated B content of some foods [23], [24].

The biological plausibility for the chemopreventative effect of BA is supported by several lines of investigation. In immunocompromised mice, BA supplementation reduced the growth of transplanted human prostate tumors, decreased IGF-1 tissue concentrations, and lowered serum prostate specific antigen levels [25]. In cell culture, BA reduced the proliferation of human cancer prostate cell lines in a dose dependent manner and inhibited cell migration and invasion [16], [26], [27]. We discovered the relationship between BA's ability to inhibit prostate cancer cell proliferation and calcium signaling after mass spectrometry studies showed the affinity of BA for NAD^+^ was reduced by phosphorylation and therefore potentially subject to biological regulation [28], [29]. BA was also shown to be a non-competitive inhibitor of ADP-ribosyl cyclase [30]. This led us to study its impact on the NAD^+^/cADPR Ca^2+^ release pathway. We showed that pharmacological levels of BA (250 and 1000 µM), but not methylboronic acid, decreased NAD^+^ stimulated release of Ca^2+^ without affecting calcium release when applied on its own [27]. It was not clear from these studies if BA was inhibiting Ca^2+^ release by interfering with NAD^+^ conversion to cADPR, blocking release of Ca^2+^ stores, or interfering with CCE. It also raised the possibility that BA concentrations in the normal blood range might be able to inhibit Ca^2+^ release from ryanodine receptor (RyR) sensitive stores. Here we demonstrate that physiological levels of BA inhibit Ca^2+^ release from RyR responsive stores in human prostate epithelial cells and lower luminal Ca^2+^ levels.

## Methods

### Cell Cultures

Experiments used prostate cancer cell lines DU-145 and LNCaP and the non-tumor cell line PWR1E. Cells were grown and maintained in cell culture plates at 37°C in 95% air and 5% CO_2_ humidified incubator. For confocal experiments, cells were cultured on glass cover slips for 24 hours prior to performing assays and studied at a confluency of less than 80%. DU-145 and PWR1E cells were acquired from American Type Culture Collection (ATCC, Manassas, VA) and LNCaP was a gift from Dr Allen Pantuck of the Department of Urology, Geffen School of Medicine at the University of California, Los Angeles. RPMI cell culture media was supplemented with 10% fetal bovine serum (FBS), penicillin (100 U/ml), streptomycin (100 µg/ml), and L-glutamine (200 mM). PWR1E non-tumor cells were maintained in Keratinocyte media containing streptomycin (100 µg/ml), L-glutamine (200 mM), 2.5 µg human recombinant EGF, and 25 mg of bovine pituitary extract (Gibco, Carlsbad, CA). Boron depleted media was prepared using the boron specific ionic exchange resin, Amberlite IRE-743 (Sigma) as previously described [16] and modified as follows. Nine grams of autoclaved IRA-743 ion exchange resin were added to 500 ml media and mixed on an Orbit rotator at 75 to 100 rpm for 15 to 20 hrs at 9°C.

### Measurement of Ca^2+^ Transients

**Figure 1:**
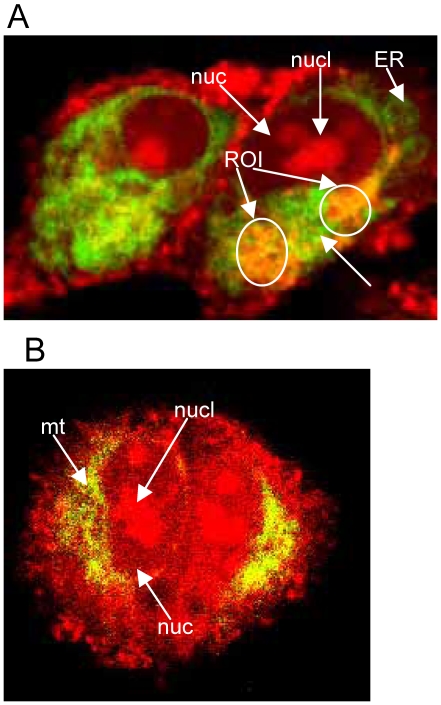
DU-145 cells demonstrating fluorescently labeled stored calcium, ER and mitochondria. A. Two DU-145 cells labeled with red fluorescent intracellular calcium fluorophore, Rhod-2, and green fluorescent endoplasmic reticulum label, ER tracker. Yellow indicates overlap of Ca^2+^ and ER and the circle is the region of interest (ROI) analyzed in these experiments. Arrows indicate organelles: nucl (nucleolus), nuc (nucleus), ER (endoplasmic reticulum) B. Two DU-145 cells labeled with red fluorescent intracellular Ca^2+^ fluorophore, Rhod-2, and green fluorescent mitochondrial label, Mito tracker. Yellow indicates overlap of calcium and mitochondria. Arrows indicate organelles: nucl (nucleolus), nuc (nucleus), mt (mitochondria).

Storage Ca^2+^ changes were monitored using the calcium sensitive dye Rhod-2, AM ester (Biotium, Hayward, CA) which accumulates in organelles. Rhod-2, AM ester has a Kd of 1 mM and has been shown to compartmentalize, particularly in the mitochondria, but as we show, in other organelles as well (Fig. 1). We also used ER Tracker green and Mito Tracker green (Molecular Probes, Carlsbad, CA) in conjunction with Rhod-2, Am ester. They are highly specific fluorescent labels for the endoplasmic reticulum and mitochondria, respectively. We analyzed Ca^2+^ changes in response to BA and various agonists by selecting regions of interest in cells that overlapped the red Rhod-2 Ca^2+^ label with the green ER tracker (Fig. 1A) [31]–[33]. Rhod-2, AM ester was prepared as a 1 mM stock solution in DMSO and diluted in either complete RPMI media or complete Keratinocyte media to 3 µM. Cells were incubated with Rhod-2 AM ester (5 µM) and ER or Mt Tracker (0.5 µM, 250 nM respectively) in RPMI or Keratinocyte media for 30 minutes at 37°C. Ca^2+^ release was stimulated using agonists in combination with different concentrations of BA in Ringers solution. Images were collected with a Zeiss 510 LSM 5 Pascal mounted to an upright microscope (Zeiss Axioplan 2) equipped with an Axoplan X63 (NA 0.95) water immersion objective. A HeNe laser was used to excite Rhod-2 at 543 nm. 488 nm from a laser diode was used to excite ER or Mt Tracker. The emission was collected on a photomultiplier tube through a 560 nm LP filter for Rhod-2 and a 505 LP filter for ER and Mito trackers. Additional magnification, time series, and background subtraction were controlled using Zeiss LSM acquisition software. All images were acquired as 12 bit.

### Analysis of Ca^2+^ release using confocal microscopy

BA treatments were applied in Ca^2+^ free Ringer's solution prepared using ultrapure water to remove B [16], [34]. Ca^2+^ release from the RyR was activated by the addition of either 25 µM cADPR to 10 µM digitonin permeabilized cells, or 20 mM caffeine or 100 µM 4-chloro-m-cresol (4cmc) in intact cells [35], [36]. Inhibition of Ca^2+^ release was achieved using 10 µM dantrolene or varying concentrations of BA in the presence of an agonist [37]. SERCA was inhibited using 10 µM cyclopiazonic acid (CPA) [2]. All drug applications were for a 30 second duration in calcium free Ringer's solution (143 mM NaCl, 5 mM KCl, 1 mM MgCl_2_, 10 mM glucose, 10 mM HEPES, 1 mM EGTA-2H_2_O, 1 mM EGTA) in order to view Ca^2+^ release from the stores without input of external Ca^2+^. Drug application was followed by a three minute wash in Ringer's solution containing calcium (140 mM NaCl, 5 mM KCl, 2 mM CaCl_2,_ 1 mM MgCl_2_, 10 mM glucose, 10 mM HEPES). Solutions were delivered to the perfusion chamber at a rate of 1 ml/min by using a single-pass, gravity-feed perfusion system. Confocal images were taken every second and began 2 minutes prior to the first treatment application in order to establish a base line of fluorescence intensity. The change in fluorescence intensity (f) caused by the drug application was compared to an averaged baseline measurement which consisted of three measurements prior to the application of the drug (fo). All measurements were normalized to the base line in this manner using the ratio f-fo/fo. Typically 6–10 cells were analyzed in one field of view and all experiments were performed on a minimum of three independent preparations. The order of drug application was based on an initial experiment involving consecutive agonist-only applications. If the 2^nd^ application of agonist caused a lower Ca^2+^ release than the 1^st^, cells were treated with agonist plus BA first. This avoided the possibility that measured inhibition of Ca^2+^ release due to BA was in part due to refractory cells made so by prior treatment with agonist.

### Analysis of stored calcium levels

DU-145 prostate cancer cells were treated with BA for 1 to 72 hrs in media stripped of boron using Amberlite IRA-743 ion-exchange resin (Sigma-Aldrich, St. Louis, MO) as previously described. Just prior to analysis cells were removed from the plate using 1% trypsin digestion, centrifuged, and labeled for 45 minutes in standard media with 3 µM Rhod-2 AM ester for storage Ca^2+^ analysis. In order to monitor cytoplasmic Ca^2+^ levels, cells were labeled with 5 µM Indo-1 AM (Invitrogen, Carlsbad, CA) for 45 minutes. Labeled cells were then washed and resuspended in 1 mL of normal media. Fluorescence was analyzed using a Beckton Dickinson BD-LSR I analytic flow cytometer. Rhod-2 excitation was achieved with a 514 nm argon laser and analyzed in the (581 nm) FL-2 channel. Indo-1 was excited with a UV laser (351 nm) and analyzed on 400 nm (FL5) and 510 nm (FL4) band-pass filters. Results for Indo-1 labeled cells are given as ratios of fluorescence (FL5/FL4). Forward and side scatter were used to gate out cellular fragments [38]. Raw flow cytometry data was analyzed using FLOWJO software (Treestar Software, San Carlos, CA, USA). Results are presented as% Ca^2+^ levels compared to untreated cells. Storage Ca^2+^ levels were also analyzed in BA treated and non-treated cells by comparing Ca^2+^ storage emptying in response to 100 µM thapsigargin using confocal microscopy [39].

### Statistical analysis of the data

Data are presented as mean±SD and were analyzed using Student's paired or unpaired t-test or one-way ANOVA for multiple comparisons with Dunnett's multiple comparison post-hoc test using Graphpad Prism 4.0. A p value<0.05 was considered to be statistically significant. The equation for non-linear one site binding (hyperbola) Y = Bmax*X/(K_d_+X) was used to calculate K_d_ and Bmax values using Graphpad software.

### Chemicals and supplies

B was removed from water and media using methods previously described [13]. Caffeine, 4-CmC, ATP, CPA, dantrolene, thapsigargin, cADPR, digitonin and boric acid were obtained from Sigma (St. Louis, MO) diluted in calcium free Ringer's Solution to ensure that if DMSO was present levels were no greater than 0.1%. All solutions were prepared fresh prior to perfusion. Superfusion with 0.1% DMSO in calcium free Ringer's solution did not affect ER Ca^2+^ levels or imaging responses.

## Results

The objective of the study was to determine if physiological concentrations of BA inhibit the release of Ca^2+^ from RyR sensitive stores in human prostate cancer and non-tumor epithelial cells. DU-145 cells were loaded with Rhod-2 and ER tracker or Mito tracker to determine if Rhod-2 compartmentalized in one or both compartments. It was not possible to delineate Ca^2+^ signaling from the ER versus mitochondria in live cells using confocal microscopy. Rhod-2 labeled Ca^2+^ in mitochondria, ER, nucleolus, and other areas in the cell (Fig. 1A–B). In our experiments, a combination of ER Tracker and Rhod-2 were used to define our regions of interest on sites of stored Ca^2+^ (Fig. 1A). This area included Ca^2+^ stores located in the ER and mitochondria (Fig. 1A–B). Application of BA from 0.1–1000 µM alone did not demonstrate an immediate measurable effect on storage Ca^2+^ using confocal microscopy (data not shown).

### Boric acid inhibits Ca^2+^ release in response to ryanodine receptor agonists

**Figure 2:**
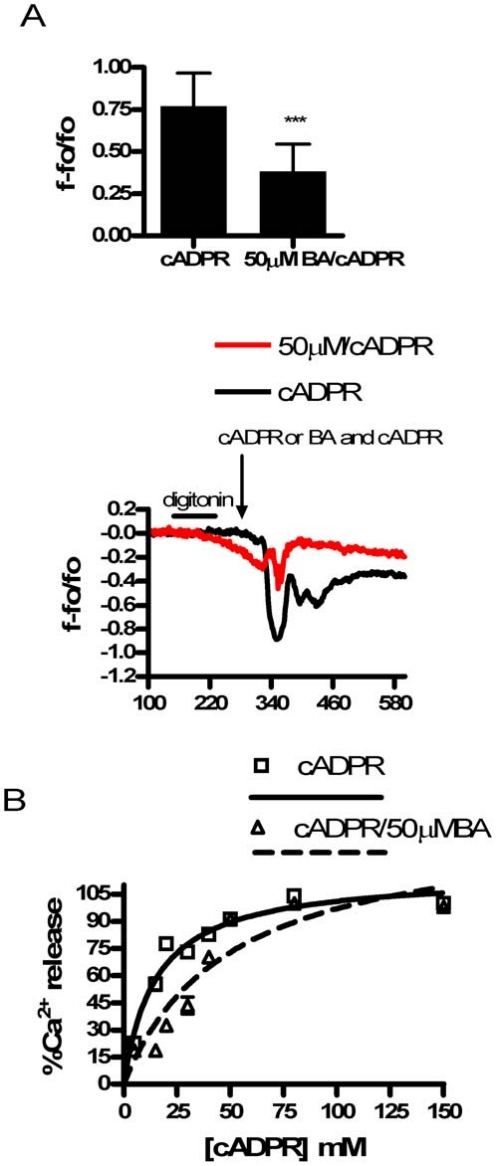
Calcium release in permeabilized DU-145 cells in response to cADPR with and without 50 µM BA present. A. 50 µM BA inhibited 25 µM cADPR induced Ca^2+^ release (n = 6; ***p<0.001). B. Competitive ligand binding study showed BA was a surmountable competitive antagonist of cADPR in a single site model. Concentrations of BA were held at 50 µM. At low cADPR concentrations BA shifted the Ca^2+^ release response to the right, but this was reversed at higher cADPR concentrations and the maximum response (Bmax) was not changed. Each data point is the mean of n = 6. R^2^ is 0.9155 and 0.8606 for cADPR without and with BA, respectively.

To determine if BA inhibited Ca^2+^ release from the RyR, we treated DU145 cells with three different RyR agonists in combination with BA. Competitive ligand binding analysis showed BA was a single site reversible competitive inhibitor of cADPR, an endogenous agonist of RyR (Fig. 2A–C). The equilibrium dissociation constant, (K_D_) for cADPR was 15.10, but in the presence of BA increased to 49.39. Inhibition by BA was reversed by increasing the concentration of cADPR and the presence of BA did not change the maximum number of binding sites (Bmax) (Table 1).

**Table 1:** Competitive Ligand Binding Study of cADPR and Boric Acid on Ryanodine Receptor Ca^2+^ Release.

	**Best Fit Values**	
Parameter	cADPR	cADPR+BA
Bmax	116.4±3.5^a^	144.8±11.9
K_d_	15.1±1.6	49.4±9.1
	**95% Confidence**	
Bmax	109.3 to 123.4	120.8 to 168.8
K_d_	11.9 to18.3	31.0 to 67.8
	**Goodness of Fit**	
Degrees of Freedom	41	40
R^2^	0.9155	0.8606
Absolute Sum of Squares	2522	6745

a Standard error of mean.

**Figure 3 pone-0006009-g003:**
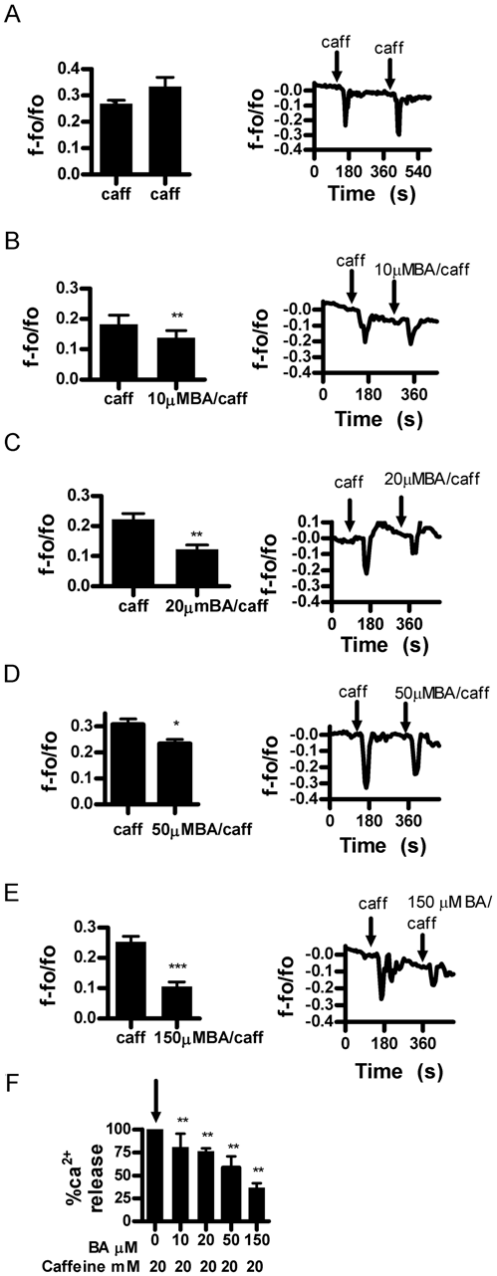
Boric acid inhibits Ca^2+^ release in response to ryanodine receptor agonist caffeine in DU-145 cells. A. Ca^2+^ release in DU-145 cells in response to consecutive applications of 20 mM caffeine (n = 6, *p = 0.0168). B. 10 µM BA significantly inhibited Ca^2+^ release (n = 6, **p<0.01). C. 20 µM M BA significantly inhibited Ca^2+^ release (n = 6, *p = 0.0163). D. 50 µM BA significantly inhibited Ca^2+^ release (n = 6, **p = 0.0042). E. 150 µM BA significantly inhibited Ca^2+^ release, n = 6, ***p = 0.0001. F. combined data analysis showing inhibitory dose response effect of BA on caffeine stimulated Ca^2+^ release, (n = 6 per concentration, **p<0.01). Figures 2–9, each bar represents the mean response of 6 experiments (n = 6) replicated 3 times. The line scans on the right of each bar graph are representative responses from a single experiment.

**Figure 4 pone-0006009-g004:**
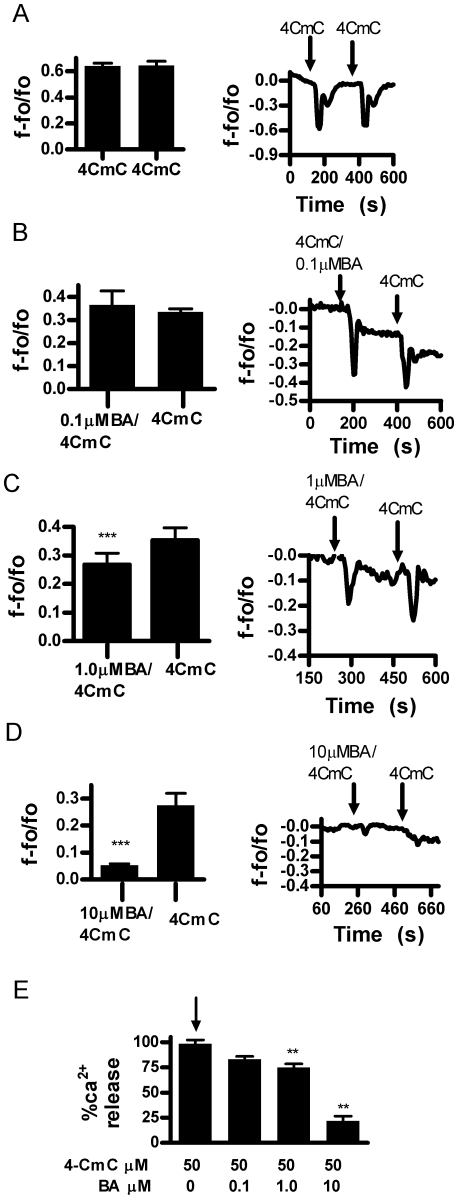
Boric acid inhibits Ca^2+^ release in response to ryanodine receptor agonist 4-CmC (50 µM) in DU-145 cells. A. Consecutive applications of 4-CmC did not alter the calcium release response (n = 6, NS). B. 0.1 µM BA did not inhibit Ca^2+^ release. C. 1.0 µM BA inhibited Ca^2+^ release (n = 6, *p<0.05). D. 10 µM BA inhibited Ca^2+^ release (n = 6, ***p = 0.0005). E. Combined data analysis showing a dose dependent Ca^2+^ release response (n = 6 per concentration (**p<0.01).

We then analyzed the effect of BA on other RyR agonists in intact cells. We first used caffeine [20 mM] an agonist that potentiates RyR sensitivity to its native ligand, Ca^2+^. In response to two consecutive applications of caffeine, the second release was slightly greater than the first (Fig. 3A). This sequence was then repeated with BA added in combination with caffeine in the second application. The results of these experiments show that BA reduced Ca^2+^ release in a dose dependent manner from 10 to 150 µM BA (Fig. 3B–F). We then examined the effect of BA on 4CmC, a direct agonist of the RyR. Consecutive applications of 50 µM 4CmC resulted in equivalent Ca^2+^ release (Fig. 4A). As observed with caffeine, BA decreased 4CmC stimulated Ca^2+^ release in a dose dependent manner (Fig. 4B–E). However, inhibition began at 1 µM BA and reached a maximum at 10 µM BA.

**Figure 5 pone-0006009-g005:**
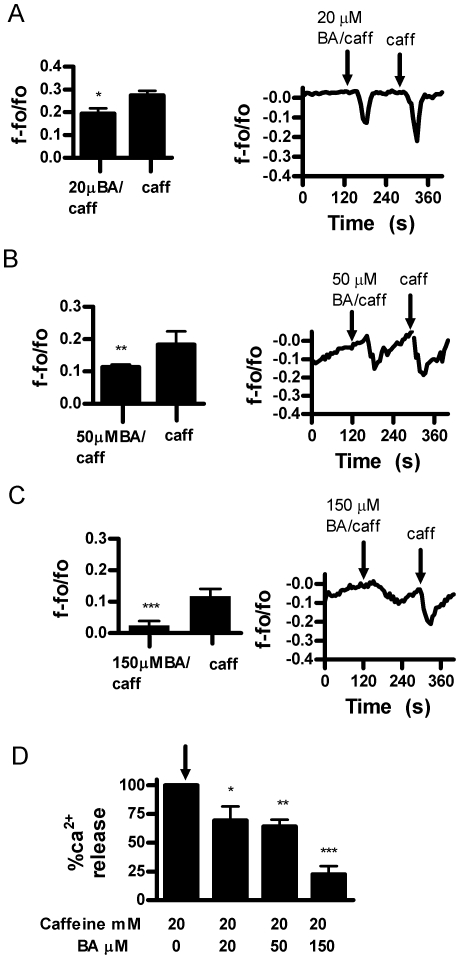
Boric acid inhibition of caffeine (20 mM) stimulated Ca^2+^ release in LNCaP cells. A. 20 µM BA inhibition of calcium release (n = 6, *p = 0.0226). B. 50 µM BA inhibition of Ca^2+^ release (n = 6, **p = 0.0019). C. 150 µM BA inhibition of caffeine induced Ca^2+^ release (n = 6, ***p<0.0001). D. Combined data showing concentration dependent inhibition of caffeine stimulated release (n = 6, **p<0.01).

**Figure 6 pone-0006009-g006:**
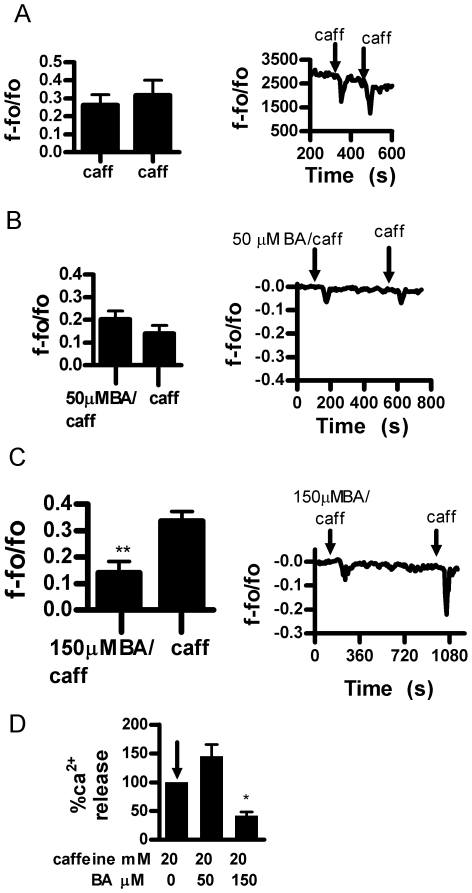
Boric acid inhibition of caffeine (20 mM) stimulated Ca^2+^ release in PWR1E non-tumor cells. A. Consecutive caffeine applications in PWR1E prostate cell. (n = 6, NS). B. 50 µM BA and caffeine induced Ca^2+^ release shows no inhibition (n = 6, NS). C. 150 µM BA inhibited caffeine induced Ca^2+^ release (n = 6, **p = 0.0029). D. Combined data showing no dose response and inhibition only at 150 µM (n = 6, *p<0.05).

Previous studies have shown that the inhibitory effect of BA on cell proliferation at the IC_50_ for DU-145 cells was approximately 10% less effective on the less aggressive LNCaP prostate cancer cell line. In addition, BA was not able to achieve a 50% reduction in proliferation in non-tumor PWR1E prostate epithelial cells in experiments using up to 4 times the IC_50_ for DU145 [16]. We examined these cell lines to determine if BA was also less effective in inhibiting caffeine sensitive Ca^2+^ release. Inhibition of Ca^2+^ release in response to caffeine in LNCaP prostate tumor cells occurred over 20 µM-150 µM BA (Fig. 5A–D). Thus, LNCaP cells were less sensitive to BA compared to DU-145. LNCaP cells were not responsive to 4CmC treatment (not shown). The PWR1E non-tumor, hyperplasic prostate cell line required 150 µM BA to inhibit caffeine stimulated Ca^2+^ release (Fig. 6A–D). PWR1E cells were non-responsive to 4CmC treatment (not shown).

### Boric acid inhibits cyclopiazonic acid induced Ca^2+^ release from the ER

**Figure 7 pone-0006009-g007:**
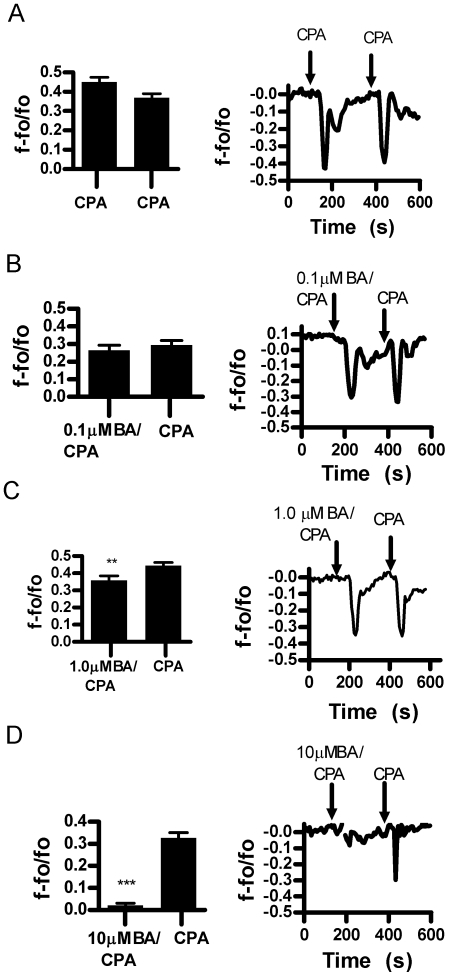
Boric acid inhibits CPA (10 µM) induced Ca^2+^ release from the ER in DU-145 cells. Cells were tested with CPA+BA followed by CPA. A. Consecutive applications of CPA did not alter Ca^2+^ release (n = 6, NS). B. 0.1 µM M BA did not inhibit CPA stimulated Ca^2+^ release (n = 6, NS). C. 1.0 µM BA inhibited CPA stimulated Ca^2+^ release (n = 6, **p = 0.0037). D. 10 µM BA inhibited CPA stimulated Ca^2+^ release (n = 6, ***p<0.0001).

**Figure 8 pone-0006009-g008:**
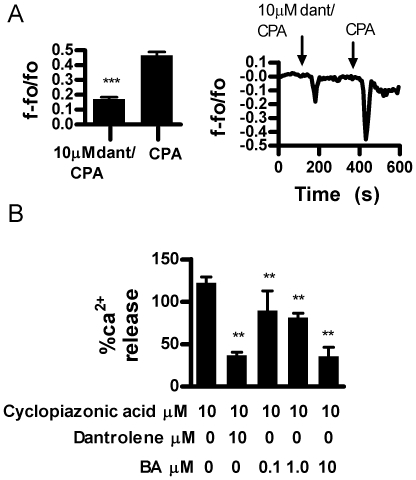
Dantrolene inhibits CPA induced Ca^2+^ release in DU-145 cells. A. Cells were tested with CPA (10 µM)+dantrolene (10 µM) followed by CPA (n = 6, ***p<0.0001. B. BA inhibited CPA stimulated Ca^2+^ release in a concentration dependent manner. The inhibitory effect of dantroline and BA were equivalent at 10 µM, CPA (n = 6, ** p<0.01).

The release of ER Ca^2+^ stores in some non-excitable cells can be stimulated using cyclopiazonic acid (CPA) which inhibits SERCA in a manner similar to thapsigargin, but can be washed out [40]. BA (0.1–1000 µM) by itself did not alter storage calcium levels during the time course of the experiment (not shown). Responses to consecutive applications of CPA [10 µM] did not decay (Fig. 7A). Simultaneous application of CPA and 1 µM BA caused a significant decrease in release and 10 µM BA caused near complete inhibition (Fig. 7B–D). We then tested the effect of dantrolene, a known inhibitor of the RyR on Ca^2+^ release by CPA. We found that 10 µM dantrolene inhibited CPA stimulated Ca^2+^ release at a level equivalent to 10 µM BA (Fig. 8A–B).

### Boric acid treatment lowers storage [Ca^2+^] with no effect on cytoplasmic [Ca^2+^]

**Figure 9:**
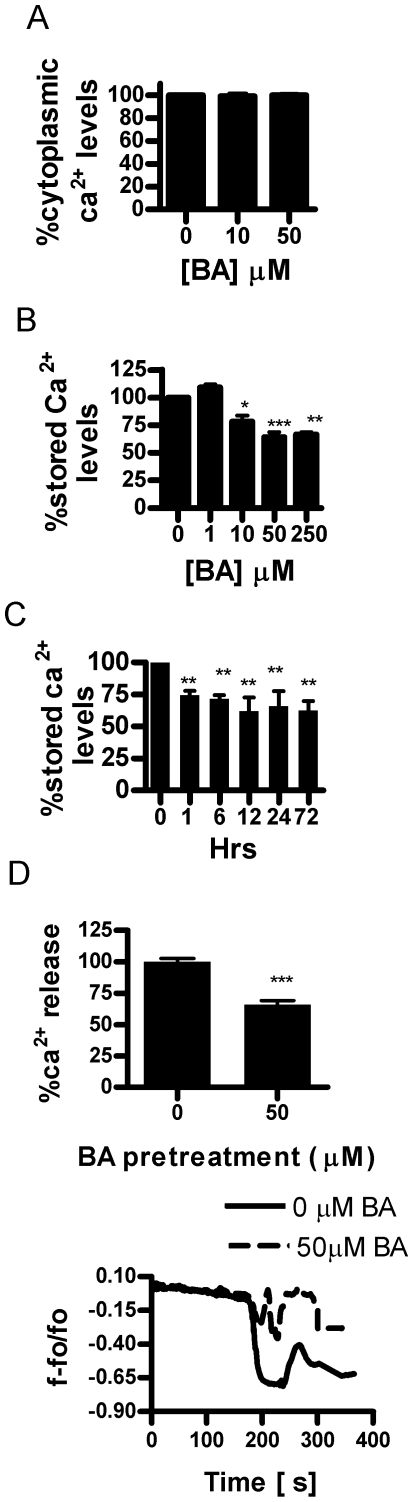
Boric acid treatment lowers luminal storage [Ca^2+^]_st_, but has no effect on cytoplasmic [Ca^2+^]_cyt_ in DU-145 cells. A. Relative [Ca^2+^]_cyt_ following exposure to 0, 10 and 50 µM BA for 24 hrs (n = 5, NS). B. [Ca^2+^]_st_ as percent of 0 treatment in DU-145 cells treated with 0–250 µM BA for 24 hrs. Boric acid [10–50 µM] reduced ER calcium levels by more than 22%–32% compared to untreated cells (n = 5,*p<0.05, **p<0.01, ***p<0.001). C. Reduction of [Ca^2+^]_st_ in DU145 cells treated with 50 µM BA from 1–72 hours (n = 5, **p<0.01). D. Confocal measurement showed pretreatment of cells with BA for 24 hrs lowered thapsigargin stimulated Ca^2+^ release compared to untreated cells (n = 6, ***p<0.0001).

Our results showing BA inhibited Ca^2+^ release lead us to analyze relative [Ca^2+^]_st_ levels using Rhod-2 stained cells and relative [Ca^2+^ ]_cyt_ levels using Indo-1 stained cells and flow cytometry. Exposure of DU-145 cells to BA [10–50 µM] for 24 hours did not affect [Ca^2+^ ]_cyt_ (Fig. 9A). However, reductions [Ca^2+^]_st_ (22%) occurred with 10 µM BA resulted in a 32% reduction in [Ca^2+^]_st_ by 50 µM BA at 1 hour (Fig. 9B). Neither higher BA concentrations nor treatment with 10 µM BA up to 72 hours resulted in further reduction in [Ca^2+^ ]_st_ (Fig. 9BC). We then conducted confocal measurements of thapsigargin stimulated Ca^2+^ release after DU-145 cells were pretreated with BA (50 µM) for 24 hrs and observed a 35% decrease in Ca^2+^ release (Fig. 9D).

## Discussion

This study reports the unexpected finding that stored Ca^2+^ release and luminal levels can be modulated by physiologically relevant levels of BA in DU-145 prostate cancer epithelial cells. This is relevant to our understanding of cancer risk since blood levels of BA are determined by the consumption of B in drinking water and plant derived foods [17]. It is likely to be the first cellular response to B exposure and thus a starting point for understanding how the risk of prostate and lung cancer is reduced by B in a dose dependent manner [16], [21], [22]. BA exhibited the attributes of a classic antagonist in that it did not have an immediate effect on Ca^2+^ release when applied by itself and its effects were agonist dependent. BA dose dependently inhibited Ca^2+^ release in response to cADPR, an endogenous agonist of the RyR, and agonists caffeine and 4-CmC (Fig. 2–5). In our competitive ligand binding analysis, BA displayed the characteristic of a single site antagonist in that it was reversible at higher concentrations of cADPR (fig. 2B). BA increased K_d_, from 15.1 to 49.4, but did not affect BMax (Table 1). BA has been shown to bind to cADPR at high concentrations, however, BA's ability to inhibit cADPR, caffeine and 4CmC induced Ca^2+^ release indicates that at physiological concentrations BA may bind to the cADPR binding site on the RyR stabilizing the Ca^2+^ channel in its inactive state [30].

Proliferation of LNCaP cells have been reported to be about 10% less sensitive and PWR1E non-tumor cells are more than 4 fold less sensitive to BA than DU-145 cells [16]. We also observed this pattern of sensitivity in BA's ability to inhibit caffeine stimulated Ca^2+^ release. The lowest effective concentration for DU-145 was 10 µM BA, LNCaP was 20 µM BA, and PWR1E was 150 µM BA (Fig. 3F, 5D & 6D). In addition to the decreased responsiveness to BA, both LNCaP and PWR1E were non-responsive to 4CmC treatment. RyR isoforms 1 and 2, but not 3 are known to be activated by 4CmC in some cell lines [41]. It is possible that cell line differences were due to differences in RyR isoforms or their expression. LNCaP cell lines have been shown to express RyR 1 and 2, but not 3 [42]. We were unable to locate studies in the literature that identified RyR isoforms in DU-145 or PWR1E cells in any prostate cell line.

Renal function tests have shown BA is reabsorbed by the kidney in non-pregnant and pregnant women [43]. The discovery of an electrogenic, voltage-regulated bicarbonate sodium-coupled borate co-transporter (NaBC1) in rat kidney tubules may explain this observation, but it has not yet been confirmed by another laboratory. NaBC1 expression induced proliferation in HEK and Hela cells by activation of the MAPK pathway 16 hours post-treatment [13]. It is unknown at this time whether NaBC1 is expressed in prostate tumor and non-tumor cell lines, but differences in its expression has been raised as a possible explanation for the variation in cellular sensitivity to BA between prostate tumor and non-tumor cells [10].

To further explore BA's ability to inhibit stored Ca^2+^ release we tested its effects in the presence of CPA (Fig. 7–8). CPA inhibits the SERCA channel resulting in emptying of Ca^2+^ stores [44]. Our study showed that BA dose dependently blocked CPA mediated Ca^2+^ release in DU-145 cells. We also observed a reduction of storage Ca^2+^ levels by 22% to 32% in 10 to 50 µM BA treated DU-145 cells compared to untreated cells. STIM proteins are involved in triggering Ca^2+^ influx into the ER [45]–[48]. If BA reduces Ca^2+^ leakage through RyR channels the major loss by leakage may occur through presenilins and other non-channel proteins that do not stimulate STIM proteins. This would result in decreased stored Ca^2+^ levels that are unable to signal refilling.

The importance of Ca^2+^ in cell cycle control and proliferation is a well established area of cancer research, but it has not been studied as a mode of action in cancer prevention [49]–[51]. BA's ability to modulate cell proliferation has been linked to changes in the expression of cyclins and the MAPK pathway in DU-145, HEK293 and HeLa cells [13], [16], [26]. However, it is possible these effects occur in response to reductions in Ca^2+^ release or storage. In the prostate cancer LNCaP cell line, Humez observed that IGF (5 ng/ml), which increases cell growth, increased ER [Ca^2+^]_ER_, whereas TNF alpha (1 ng/ml), which reduces cell proliferation, reduced [Ca^2+^]_ER_ [52].

In conclusion, BA has previously been reported to reduce the risk of prostate cancer in humans and reduce tumor growth and prostate cancer cell proliferation, migration, and invasion. Our results demonstrate that physiological levels of BA inhibit agonist stimulated release of stored Ca^2+^ in a dose dependent manner and lower stored Ca^2+^ storage levels. This suggests that BA's ability to dampen Ca^2+^ signaling in cancer cells underlies its ability to reduce clinical cancer risk.
